# Molecular Evolution and Mechanisms of Plants NRAMP Transporters in Response to Heavy Metal Stress

**DOI:** 10.3390/plants15101582

**Published:** 2026-05-21

**Authors:** Li Hao, Jingjing Chen, Mazarin Akami, Cabrel Bafong Ngueya, Diane Pocssie Samenoug, Haiyang Tang, Qianqian Tang, Qingfeng Zheng, Yiling Peng, Yanli Zhang, Fuhui Rong, Jin Wu, Rongsen Wang, Chenchen Zhao, Xiaojian Wu, Wei Jiang

**Affiliations:** 1 MARA Key Laboratory of Sustainable Crop Production in the Middle Reaches of the Yangtze River (Co-Construction by Ministry and Province), College of Agriculture, Yangtze University, Jingzhou 434025, China; haoli.st@yangtzeu.edu.cn (L.H.); 2022710756@yangtzeu.edu.cn (J.C.); 2023710782@yangtzeu.edu.cn (H.T.); 2023710801@yangtzeu.edu.cn (Q.T.); 2022710786@yangtzeu.edu.cn (Q.Z.); 2024710821@yangtzeu.edu.cn (Y.P.); zhangyanli.st@yangtzeu.edu.cn (Y.Z.); rongfh.stu@yangtzeu.edu.cn (F.R.); 2025720926@yangtzeu.edu.cn (J.W.); 2024710830@yangtzeu.edu.cn (R.W.); 2 Department of Biochemistry, University of Douala, Douala P.O. Box 24157, Cameroon; akami@xhlab.ac.cn; 3 Tasmanian Institute of Agriculture, University of Tasmania, Launceston, TAS 7250, Australia; ngueyacabrel@gmail.com (C.B.N.); poccsiediane@gmail.com (D.P.S.); chenchen.zhao@utas.edu.au (C.Z.); 4 Xianghu Laboratory, Hangzhou 311231, China; 5 College of Agricultural, Nanjing Agricultural University, Nanjing 210095, China

**Keywords:** NRAMP, heavy metals, plant, uptake, transport, stress response, detoxification, gene family, soil remediation

## Abstract

Heavy metals in the soil inhibit plant growth, which significantly reduce the crop yield and quality. Natural Resistance-Associated Macrophage Proteins (NRAMP) are widely distributed on the plasma and vacuolar membranes of plant roots, stems, and leaves. The *NRAMP* gene family plays a crucial role in modulating plant heavy-metal uptake, sequestration, distribution, and translocation, while the molecular evolution and mechanisms underlying these processes remain unclear. Here, we reviewed recent progress on plant *NRAMP* genes, focusing on their structural characteristics and functions in the absorption, transport, accumulation, and detoxification of various heavy metals. Furthermore, we performed an evolutionary analysis of *NRAMP* in green plants, indicating expansion and tandem duplication in ferns. In addition, their key amino acid sequences and secondary structures were highly conserved across plant species. The expression of diverse tissue showed that *NRAMP* genes displayed distinct spatial regulation in the leaves and roots. We also explored the underlying molecular mechanisms and regulatory pathways by which *NRAMP* genes influence heavy metal uptake. Therefore, by integrating structural conservation, molecular evolution, tissue- and single-cell expression patterns, ion-stress-responsive expression, regulatory pathways, and the Cd–Mn nutrient–toxin trade-off, this review provides a framework for identifying unresolved *NRAMP* functions and for guiding future strategies in low-heavy-metal crop breeding, metal homeostasis engineering, and phytoremediation.

## 1. Introduction

Plant growth depends on root systems for absorption of mineral nutrients from soil. During this process, non-essential heavy metals can also be taken up, leading to potential toxicity [1]. In China, approximately 25% of arable land is polluted with heavy metals in varying degrees, posing a significant threat to food production, safety, and public health [2]. Therefore, it is important to research the functions of plant metal transporters [3,4]. Exploring the mechanism of plants acquiring essential metal elements while mitigating the toxicity of non-heavy-metal elements helps uncover the mechanisms of selective uptake and detoxification [5,6], which is fundamental in improving the safety of agricultural products [7].

Plants possess diverse families of metal transporters in the uptake, translocation, and accumulation of metal ions [8,9,10,11,12,13,14]. These proteins include Natural Resistance-Associated Macrophage Proteins (NRAMPs) [10,11,14], Cation Diffusion Facilitators/Metal Transporters (CDF/MTPs) [12], Heavy Metal ATPases (HMA) [13,14], Yellow Stripe Transporters (YSLs) [15], Calcium/Cation Antiporters (CaCAs) [16], ATP-Binding Cassette Transporters (ABCs) [17], Bivalent Cation Transporters (BICATs) [18], and Vacuolar Iron Transporters (VITs) [19,20]. Among them, the *NRAMP* gene family are primarily involved in the acquisition, transport, and homeostasis of divalent metal ions in plants, such as iron (Fe), manganese (Mn), cadmium (Cd), lead (Pb), and mercury (Hg) [21]. The expression patterns, subcellular localizations, and metal-transport functions of *NRAMP* genes exhibit considerable variation across plant species, and this apparent contrast between structural conservation and functional diversification can be interpreted as the result of a conserved transport scaffold combined with lineage- and tissue-specific regulatory divergence [22,23,24]. Most plant NRAMPs retain conserved transmembrane domains, metal-binding residues, and NRAMP/SLC11 transport motifs, suggesting that the basic capacity for divalent metal transport has been maintained during plant evolution [25]. However, gene duplication, promoter diversification, differences in *cis*-acting elements, subcellular targeting signals, and stress-responsive transcriptional regulation can alter where, when, and how individual NRAMP members function [26]. Therefore, the conserved structure of NRAMPs can acquire distinct physiological roles in different species, tissues, and environmental contexts, including root uptake, vacuolar remobilization, xylem/phloem redistribution, seed loading, and heavy-metal detoxification [9].

Recent genome-wide studies further support the evolutionary conservation and functional importance of *NRAMP* genes in plant metal transport and stress tolerance. In *Arabidopsis thaliana*, identified six *AtNRAMP* genes. They showed that conserved NRAMP transmembrane domains, stress- and hormone-responsive *cis*-elements, and differential expression under heavy-metal and phytohormone treatments support their roles in metal homeostasis and stress adaptation. In *Aegilops tauschii*, [27] 11 AetNRAMP proteins were found, which classified them into 5 subgroups, and showed that several *AetNRAMP* genes respond to heavy-metal stress. In addition, functional validation further suggested roles for *AetNRAMP1* in Cu transport and for AetNRAMP3 in Mn-stress responses. In the woody model plant *Populus trichocarpa*, [28] 11 *PtNRAMP* genes were identified, and they differentially respond to Fe, Mn, Zinc (Zn), and Cd stresses, which demonstrated that several *PtNRAMPs* can mediate Cd, Mn, or Fe transport in yeast. In total, these studies reinforce the view that *NRAMP* genes are evolutionarily conserved metal transporters with lineage-specific regulatory and functional diversification [27,28,29].

Recent studies and reviews have substantially advanced our understanding of plant NRAMP transporters, especially their roles in Cd and Mn transport, metal homeostasis, and low-Cd crop breeding [25,30,31]. For example, NRAMPs are now recognized as common transporters for Cd and Mn, making them central to the nutrient–toxin trade-off in crops [25,32]. Recent reviews have also emphasized that NRAMP contribution to Cd accumulation depends strongly on substrate specificity, tissue expression, and subcellular localization [31], while NRAMPs have emerged as important targets for grain-safety breeding and metal-homeostasis engineering [28]. However, several important questions remain less understood, including how conserved structural features translate into plant-specific transport activity, how NRAMP regulatory mechanisms differ among monocots, dicots, and early-diverging lineages, how tissue- and single-cell expression patterns relate to metal allocation, and how NRAMP alleles can be manipulated to reduce toxic metal accumulation without disrupting essential micronutrient homeostasis. Therefore, this review differs from previous summaries by combining functional evidence with comparative evolutionary analysis, tissue-specific and single-cell expression profiles, ion-stress expression responses, and regulatory mechanisms to provide a broader platform for future NRAMP research.

Here, we not only summarized the structural characteristics, transport functions, and regulatory pathways of plant NRAMPs under heavy-metal stress, but also integrated comparative evolutionary and expression analyses across algae, bryophytes, ferns, gymnosperms, and angiosperms. By combining phylogenetic conservation, motif variation, gene duplication patterns, tissue- and single-cell expression profiles, and ion-stress response data from rice (*Oryza sativa*) and *A. thaliana*, this review provided a broader evolutionary and functional framework for understanding NRAMP-mediated metal homeostasis and for guiding future strategies in low-heavy-metal crop breeding and phytoremediation.

## 2. Plant NRAMP Gene Family

### 2.1. Distribution of NRAMP Genes in Plant Genomes

NRAMPs are integral membrane transporters. Initially identified in rat intestines for their role in divalent metal ion uptake and transport, NRAMP homologs have since been found in plants, bacteria, fungi, and insects. It is hypothesized that the *NRAMP* family diversified prior to the emergence of eukaryotes [33]. Throughout plant evolution, the *NRAMP* gene family has undergone duplication and diversification, giving rise to distinct subfamilies that have adapted to fulfill varied biological roles and respond to environmental changes [34].

In recent years, *NRAMP* genes have been characterized in numerous plants, including *O. sativa*, *A. thaliana* [29], turnip (*Brassica rapa*) [26], Mediterranean cabbage (*Brassica oleracea*) [26], soybean (*Glycine max*) [35], cucumber (*Cucumis sativus*) [36], potato (*Solanum tuberosum*) [37], tomato (*Solanum lycopersicum*) [38], tobacco (*Nicotiana tabacum*) [14], *A. tauschii*, and black poplar (*P. trichocarpa*) [28,39]. These genome-wide studies show that *NRAMP* gene families generally retain conserved transmembrane domains and metal-transporter motifs, while differences in gene number, promoter *cis*-elements, expression patterns, and stress responses indicate species-specific regulatory diversification [27,28,29]. These studies have demonstrated that *NRAMP* genes are widely distributed across both dicots and monocots plants. Based on amino acid sequence homology, plant NRAMPs can be classified into two primary subfamilies that differ in molecular weight, sequence length, isoelectric point, and exon number [22,23,40]. Furthermore, with the development of omics [41,42], the potential roles of many *NRAMP* genes have been elucidated in response to diverse heavy metal stresses, while the fine roles need to be explored through gene editing in distinct plants [43].

### 2.2. Structure of Plant NRAMPs

NRAMPs are membrane-integrated peptides containing 10 to 12 amino acids. Most atomic-level knowledge of NRAMP conformational changes currently comes from bacterial SLC11/NRAMP homologues rather than plant NRAMPs. Structural studies of prokaryotic NRAMPs, including ScaDMT from *Staphylococcus capitis* and DraNramp from *Deinococcus radiodurans*, have revealed a conserved LeuT-like fold and a central metal-binding site that coordinates transition metals such as Mn^2+^, Fe^2+^, and Cd^2+^ [10,44]. Subsequent high-resolution structures of DraNRAMP captured multiple conformational states, including outward-open, inward-open, and occluded (inward-occluded) states, supporting an alternating-access transport model coupled to proton movement [32,45]. More recently, high-resolution DraNRAMP structures with bound Mn^2+^ and Cd^2+^ showed that global conformational transitions are associated with changes in metal-coordination geometry and conserved polar-residue networks, providing a useful framework for understanding substrate selectivity in the NRAMP family [46,47].

However, these bacterial structural models should be applied to plant NRAMPs with caution. Although conserved motifs and predicted transmembrane architecture suggest that plant NRAMPs may share the general SLC11/NRAMP transport fold, plant NRAMPs differ in subcellular localization, tissue-specific expression, regulatory motifs, and physiological substrates. In plants, experimental evidence for NRAMP function mainly comes from gene knockout or overexpression, heterologous transport assays, subcellular localization studies, and allelic or site-directed mutational analyses. For example, OsNRAMP5 has been experimentally shown to mediate Mn and Cd uptake in rice; OsNRAMP1 contributes to Cd and Mn uptake; OsNRAMP2 participates in vacuolar Fe remobilization and Cd distribution to grains; and AtNRAMP1, AtNRAMP3, and AtNRAMP4 are involved in Mn or Fe transport and intracellular remobilization [48,49,50,51,52,53]. Mutational studies also support the functional importance of conserved residues in plant NRAMPs; for instance, the OsNRAMP5-Q337K weak allele reduces Cd accumulation while avoiding severe Mn deficiency, indicating that subtle structural changes can modify transporter activity in plants [54,55].

A major limitation in the current field is the lack of experimentally determined crystal structures of canonical plant NRAMP transporters. To date, no crystal structure or substrate-bound high-resolution structure has been reported for typical plant divalent metal NRAMPs such as OsNRAMP5, OsNRAMP1, AtNRAMP1, AtNRAMP3, or AtNRAMP4. A cryo-EM structure has been resolved for SiNRAT, a plant NRAMP-related aluminum transporter from *Setaria italica*, and this structure revealed an occluded conformation closer to an inward-facing than an outward-facing state [56]. However, SiNRAT belongs to a specialized NRAMP-related Al^3+^ transporter branch, and its structure was obtained without added metal ions; therefore, it cannot fully explain the conformational cycle, substrate-bound states, or proton-coupling mechanism of canonical plant NRAMPs involved in Cd, Mn, and Fe transport [56]. Future structural studies using cryo-EM, crystallography, AlphaFold-assisted modeling, molecular dynamics, and plant NRAMPs captured in different substrate-bound states will be essential to determine whether the bacterial alternating-access model is conserved in plants or modified by plant-specific regulatory and substrate-recognition features.

## 3. Interactions and Mechanisms Between Plant NRAMP Genes and Soil Heavy Metals

### 3.1. Functions, Conservation, and Regulatory Mechanisms of the NRAMP Gene Family in Cadmium Accumulation in Plants

Soil pollution with heavy metals such as Cd can interfere with root system development and plant growth [20]. Research has shown that members of the *NRAMP* gene family exhibit varying effects on Cd accumulation across different plant tissues. For example, in rice (*OsNRAMP1*), the gene is primarily expressed in roots and leaves and encodes a plasma membrane-localized protein [53,57]. It promotes Cd transport to the aerial parts of the plant through high expression in roots [53,58]. Its localization in mesophyll cells and parenchyma cells of leaf sheaths indicates its role in Cd uptake in these tissues [57]. Compared to the highly homologous *OsNRAMP5*, knocking out *OsNRAMP1* alone has a smaller impact on Cd absorption, while knocking out both genes significantly reduces Cd uptake. This suggests that *OsNRAMP1* and *OsNRAMP5* play complementary roles in Cd absorption in rice, with *OsNRAMP5* being more critical [50]. Additionally, overexpression of *OsNRAMP5* has been shown to reduce Cd accumulation in rice grains [59]. In contrast, disruption of its expression increases Cd uptake in roots and stems [52]. Knocking out *OsNRAMP5* also markedly lowers Cd concentration in grains (<0.05 mg kg^−1^) [16]. Precisely because *OsNRAMP5* function is closely linked to Cd accumulation, it serves as a promising candidate for Cd phytoremediation and a potential target for breeding low-Cd rice varieties. Subsequent studies identified a quantitative trait locus (QTL) containing two copies of *OsNRAMP5* that contributes to the low Cd accumulation trait in the rice variety Pokkali, further confirming the key role of *OsNRAMP5* in regulating Cd accumulation [60]. Furthermore, *OsNRAMP2* (candidate gene for *qCd3-2*) is considered a functional gene specifically involved in Cd transport. After Cd treatment, *OsNRAMP2* is induced in the aerial parts of high-Cd-accumulating varieties and exhibits subspecies specificity [40]. Subsequent studies confirmed that *OsNRAMP2* influences Cd transport from nutrient tissues to grains [49,51]. Compared to other members of the gene family, *OsNRAMP4* has been shown to reduce Cd content in grains by altering its distribution within the cell [61], while the effects of *OsNRAMP3*, *OsNRAMP7*, and *OsNRAMP8* on Cd absorption and transport remain under investigation.

The function of *NRAMP* genes is highly conserved across different species. For instance, *BcNRAMP1* in pak choi (*B. rapa* var. *chinensis* ‘Shanghaiqing’) [62], *AtNRAMP6* in *A. thaliana* [63], *PcNRAMP1* in silver poplar (*Populus canescens*) [64], *NtNRAMP1* and *NtNRAMP3* in tobacco [65,66], *MtNRAMP1* in alfalfa (*Medicago truncatula*), *TcNRAMP4* in Indian mustard (*Thlaspi caerulescens*), and *StNRAMP2* in potato [37] are all involved in Cd absorption. Similarly, *VrNRAMP5* in mung bean (*Vigna radiata*) [67], *HvNRAMP5* in barley (*Hordeum vulgare*), *TaNRAMP5* in wheat (*Triticum aestivum*) [68], and *FeNRAMP5* in buckwheat (*Fagopyrum esculentum*) [69] have been implicated in Cd transport. Recent research also indicates that the third transmembrane domain (TMD3) of *BrNRAMP1* in turnip (*B. rapa*) plays a critical role in Cd transport [70]. Under Cd stress, the expression of *SgNRAMP1* and *SgNRAMP2* in *Stylosanthes guianensis* is specifically upregulated in roots and leaves. In *Brassica napus*, most *NRAMP* genes show decreased expression levels as Cd concentrations increase. In contrast, certain genes (e.g., *BnNRAMP5.1* and *BnNRAMP5.2*) are upregulated at low Cd levels but downregulated under high Cd exposure [40]. These studies suggest that *NRAMP* gene family members have complex regulatory mechanisms of expression under Cd stress. Recent reviews have further emphasized that NRAMP-mediated Cd accumulation is controlled not only by gene expression, but also by transporter localization, substrate selectivity, tissue-specific redistribution, and interactions with Mn nutrition, making NRAMPs central targets for both low-Cd crop breeding and phytoremediation strategies [25,30,31].

### 3.2. Roles of Plant NRAMP Genes in Manganese

Mn is an essential trace element for plants, and is involved in a variety of critical biological processes [71]. Research has demonstrated distinct roles for *NRAMP* family members in Mn uptake and homeostasis. In rice, OsNRAMP1 is involved in Mn uptake. *OsNRAMP3* may regulate the internal distribution of Mn by facilitating its transfer from the xylem to the phloem in basal nodes, thereby allocating it to young leaves, panicles, and root tips. Furthermore, *OsNRAMP5* and *OsMTP9* are known to act synergistically to mediate Mn absorption and transport in roots. Knocking out *OsNRAMP5* drastically reduces Mn uptake and its accumulation in grains (by over 90%), resulting in characteristic Mn deficiency symptoms [50,72]. The role of *OsNRAMP6*, which accumulates in vesicles near the plasma membrane, in intracellular and intercellular Mn transport remains unclear. Mutants of *OsNRAMP5* and *OsNRAMP6* exhibit increased susceptibility to rice blast and reduced heat tolerance during flowering, leading to yield losses of 20% to 30% [72,73]. It is speculated that this may be related to the conserved function of *NRAMPs* in sequestering Mn, analogous to their role in limiting metal nutrient availability to pathogens within macrophage phagosomes [32,51,74]. Thus, *NRAMPs* play a dual role: facilitating Mn acquisition from soil and its distribution to various tissues for growth, while also enhancing plant resistance to biotic stresses.

In *A. thaliana,* AtNRAMP1 is primarily localized to the plasma membrane of root epidermal and cortical cells, where it functions as a high-affinity Mn uptake transporter. AtNRAMP3 and AtNRAMP4 are localized in vacuolar membrane of leaf mesophyll cells, and their expression levels are not affected by Mn deficiency [75]. They are responsible for mobilizing Mn from the vacuole in mature tissues. Studies have indicated that specific residues (G61, D72, and N75) in AtNRAMP3 may be involved in regulating manganese transport, and that the protein’s N-terminal region is essential for maintaining its function [63]. In tobacco (*N. tabacum*), NtNRAMP6a and NtNRAMP6b are implicated in long-distance Mn transport, as their knockouts reduce Mn accumulation in aerial parts. However, this function requires further confirmation [76]. NRAMP-mediated Mn transport has also been reported in several non-model species, although the level of experimental support differs among species. In cocoa (*Theobroma cacao*), functional characterization in yeast showed that TcNRAMP3 and TcNRAMP5 have broad substrate specificity, including Fe^2+^ and Mn^2+^ transport, while TcNRAMP6 appears to be more specific for Mn^2+^ transport [77]. In peanut (*Arachis hypogaea*), genome-wide identification and expression analysis revealed 15 *AhNRAMP* genes, many of which are preferentially expressed in roots and are responsive to Fe deficiency and Cd accumulation, indicating their potential roles in Fe/Cd interactions rather than direct evidence for Mn uptake [78]. In *Stylosanthes guianensis*, SgNramp1 is plasma membrane-localized, complements the Mn uptake-defective yeast mutant *Δsmf1*, increases Mn accumulation in yeast, and is therefore directly involved in Mn uptake [79].

A critical consideration in manipulating *NRAMP* genes is the trade-off between reducing toxic metal accumulation and maintaining essential micronutrient homeostasis. For instance, while knocking out a single *NRAMP* gene like *OsNRAMP5* can reduce Cd content in grains, it concurrently and Mn levels, potentially causing Mn deficiency. Agronomic strategies, such as applying Mn alongside lime or using coated Mn micro-fertilizers, can help mitigate plant Cd uptake while addressing Mn needs [80,81]. A significant positive correlation between Cd and Mn absorption in wheat grains grown in contaminated soils suggests a synergistic uptake mechanism for these ions [82]. Similarly, knocking out *HvNRAMP5* in barley markedly decreases accumulation of both Mn and Cd in tissues without affecting other metals. These findings confirm that certain NRAMPs function as co-transporters for Mn and Cd, playing a crucial role in their interconnected uptake, translocation, and accumulation. Recent reviews further emphasize that manipulating Mn nutrition and NRAMP activity may provide an effective strategy for reducing Cd uptake while maintaining essential micronutrient homeostasis [21,25,30].

Because many NRAMPs, especially OsNRAMP5 in rice, transport both Cd and Mn, simply knocking out *NRAMP* genes to reduce Cd accumulation may cause unintended Mn deficiency, impaired growth, yield reduction, or increased stress susceptibility. Therefore, recent studies have shifted from complete loss-of-function strategies toward more balanced approaches that reduce Cd uptake or grain accumulation while preserving sufficient Mn nutrition. These strategies include weak-point mutations, amino acid substitutions that alter Cd/Mn selectivity, natural allelic variation, promoter- or regulatory-region editing, tissue- or cell-specific modulation of *NRAMP* expression, enhanced vacuolar Cd sequestration via HMA transporters, allele pyramiding, and agronomic Mn management. The known and emerging strategies are summarized in Table 1.

### 3.3. Roles of Plant NRAMP Genes in Other Metal Elements

Plant NRAMPs have been extensively studied for their roles in transporting a diverse array of metals. Under Fe-deficient conditions, several NRAMPs facilitate Fe uptake from soil by roots and its intracellular distribution. For instance, *SlNRAMP1* in tomato is highly expressed in the root cortex and vascular parenchyma, mobilizing Fe within the vascular tissues in response to deficiency. In *A. thaliana*, *AtNRAMP3* and *AtNRAMP4* are induced by Fe deficiency and participate in Fe^2+^ transport mobilizing vacuolar Fe stores to support early development [83], In rice, OsNRAMP2 transports Fe from vacuoles to the cytoplasm, playing a critical role during seed germination [84]. In soybean, *GmNRAMP2a* and *GmNRAMP2b* are involved in Fe trafficking and are modulated by inorganic nitrogen, influencing symbiotic nitrogen fixation [35].

*NRAMP* genes also contribute to the transport of other metals. *AtNRAMP4* in *Arabidopsis* affects Zn accumulation in roots. *KoNRAMPs* in the mangrove plant *Kandelia obovata* are involved in copper (Cu) uptake [85]. *TpNRAMP5* in Polish wheat is predominantly expressed in roots and basal stems, promoting the accumulation of Cd, Co, and Mn, while having no significant effect on Zn and Fe. *SgNRAMP2* expression is upregulated in leaves under Fe deficiency but downregulated in roots. Al and Lanthanum (La) treatments suppress the expression of *SgNRAMP1* in roots but increase *SgNRAMP2* expression. Under varying Pd concentrations, some *NRAMP* genes in *Hippophae rhamnoides* are significantly downregulated at low concentrations but upregulated at high concentrations [86]. Furthermore, *OsNRAMP4* can transport Al^3+^, indicating that *NRAMP* genes also exhibit selectivity for non-divalent metals [61].

Research has shown adequate supply of essential metals can reduce the accumulation of harmful metal ions in plants. For instance, a sufficient supply of Fe in rice can inhibit Cd accumulation, thereby improving growth and yield. Fe forms a protective layer on rice roots, reducing Cd uptake and alleviating its toxicity. However, the specific effects of metal types and concentrations on *NRAMP* gene expression require further investigation.

### 3.4. Molecular Mechanisms of NRAMP-Mediated Heavy Metal Uptake, Translocation, and Accumulation

NRAMP-mediated heavy metal accumulation can be understood as a multi-step process involving root uptake, radial transport, xylem loading, shoot redistribution, vacuolar remobilization, and final deposition in sink organs such as seeds or grains. At the root surface, plasma membrane-localized NRAMPs mediate the entry of divalent metal ions into epidermal, cortical, exodermal, and endodermal cells. In rice, OsNRAMP5 is considered a major transporter for Cd and Mn uptake, whereas OsNRAMP1 contributes to Cd and Mn uptake through a partially overlapping but non-redundant pathway [50,52,57]. Therefore, Cd entry through NRAMPs often occurs through transport systems originally evolved for essential micronutrients such as Mn and Fe, explaining the strong nutrient–toxin trade-off observed when *NRAMP* genes are knocked out or modified [21,25,54].

At the cellular level, the subcellular localization of NRAMPs determines whether they promote metal influx, vacuolar release, or intracellular redistribution. Plasma membrane-localized NRAMPs generally contribute to metal uptake from the rhizosphere or intercellular space, whereas tonoplast-localized NRAMPs can remobilize metals from vacuolar stores. For example, OsNRAMP2 is localized at the tonoplast and mediates Fe remobilization during seed germination; knockout of OsNRAMP2 significantly decreases Cd distribution from leaves and straw to rice grains [49]. Similarly, OsNRAMP2 facilitates Cd efflux from vacuoles and contributes to root-to-shoot Cd translocation and differences in grain Cd accumulation between japonica and indica rice [51]. These findings indicate that vacuolar Cd efflux is an important mechanism contributing to long-distance Cd remobilization and grain accumulation.

The molecular basis of NRAMP substrate selectivity is closely associated with conserved transmembrane domains, metal-binding residues, and conformational transitions. Structural studies have shown that NRAMP transporters undergo outward-open, occluded, and inward-open conformational states, supporting an alternating-access model for divalent metal transport [32,46,47]. High-resolution structures with bound Mn^2+^ and Cd^2+^ further revealed that these metals exhibit distinct coordination features during the transport cycle, helping explain why NRAMPs can transport both essential metals and toxic analogs [46]. In addition, conserved methionine (Met) residues in the NRAMP metal-binding site influence substrate preference; in bacterial NRAMPs, the methionine sulfur favors Cd transport, whereas mutation of this residue reduces Cd preference while maintaining transport of Mn and Fe [44]. In plants, similar structure–function relationships have been observed. For example, the OsNRAMP5-Q337K weak allele reduces Cd accumulation while avoiding severe Mn deficiency, suggesting that fine-tuning transporter activity may be more useful for breeding than complete loss-of-function mutations [54,55].

Recent studies also show that NRAMP activity is controlled at the post-translational level. In *Arabidopsis*, Mn triggers phosphorylation-mediated endocytosis of AtNRAMP1, thereby reducing transporter abundance at the plasma membrane and preventing excessive Mn uptake [48]. A Cd-specific regulatory mechanism has also been identified in which the receptor-like kinase WAKL4 interacts with and phosphorylates NRAMP1 at Tyr488, promoting NRAMP1 ubiquitination and vacuole-dependent degradation, thereby restricting Cd uptake [3,87]. These findings indicate that plants regulate NRAMP function not only at the transcriptional level, but also by controlling transporter localization, trafficking, protein stability, and degradation.

Together, these findings suggest that three interacting layers govern NRAMP-mediated heavy metal accumulation: first, transporter expression in specific tissues and developmental stages; second, subcellular localization and trafficking among the plasma membrane, endomembrane system, and tonoplast; and third, structural determinants that control metal selectivity and transport kinetics. Future studies combining structural biology, electrophysiology, cell-specific expression analysis, genome editing, and field-based ionomics will be essential to distinguish beneficial micronutrient transport from toxic heavy-metal accumulation [41,43,88].

Plant *NRAMP* genes encode proteins with conserved domains that participate in the recognition, absorption, transport, and distribution of divalent metal ions. Upon sensing heavy metal stress, plants rapidly respond by modulating the expression of relevant proteins to enhance detoxification and mitigate heavy metal toxicity [25]. *OsNRAMP1*, *SgNRAMP1* and *SgNRAMP2* are upregulated in response to Cd exposure, whereas *OsNRAMP5*, *BnNRAMP5.1* and *BdNRAMP5.2* are downregulated under high Cd concentrations [26,53,58]. Many plants regulate the transcription levels of *NRAMP* genes to produce large amounts of NRAMPs, thereby preventing excessive accumulation of heavy metal ions and reducing cellular damage [21]. Furthermore, NRAMPs could regulate the transfer of harmful metals via sequestration and efflux mechanisms, thereby limiting their excessive accumulation in plants [89]. OsHMA3 is a vacuole-localized protein that sequesters cytosolic cadmium into vacuoles, and members of the NRAMP family may also have similar functions [90]. Upregulating the *OsNRAMP5* expression in brown rice can result in 43% reduction in Cd content [25]. NRAMPs in animal macrophages exhibit divalent metal ion efflux functions that are influenced by membrane potential and conserved charged residues [32]. And NRAMP protein transport activities may rely on proton gradients (H^+^ counter-transport) and voltage in bacteria [91]. However, these mechanisms remain poorly understood in plants and require further investigation.

## 4. Regulatory Pathways of Plant NRAMP Genes in Soil Heavy Metal Uptake and Transport

### 4.1. Phytohormone Regulation

Plant hormones play a foundational role in plant growth, development, and responses to environmental stresses. Research has shown that multiple plant hormones [ethylene (ET), auxin (IAA), abscisic acid (ABA), salicylic acid (SA), and jasmonic acid (JA)] regulate the expression of *NRAMP* genes, thereby influencing plant responses to heavy metal stress. For example, in *Spirodela polyrhiza* (duckweed) [92], potato [24], Populus (black poplar) [28], and peanut [93], *NRAMP* genes are regulated by phytohormone signaling pathways.

ET signaling pathways involve core proteins such as EIN2 (ETHYLENE-INSENSITIVE 2), which share structural similarities with NRAMP metal ion transporters [94]. In rice, the membrane protein MHZ3 (MAO HU ZI 3) interacts with EIN2’s NRAMP-like domain to stabilize EIN2, suggesting that NRAMPs may regulate metal transport through ET signaling pathways. In *Arabidopsis*, the activity of *AtNRAMP3* is regulated by ET signaling, which alters its distribution between the plasma membrane and vacuolar membrane, thereby modulating the competitive uptake of Fe and Cd [84]. Similarly, *HrLNRAMP8* in sea buckthorn exhibits analogous functions [86].

IAA regulates plant responses to environmental stresses through the AUX/IAA and ARF signaling pathways. Although links between IAA signaling and metal transporter genes have been identified [95], research on the regulation of *NRAMP* genes by IAA is limited. In sea buckthorn, the promoters of *NRAMP* family genes contain auxin-responsive elements (TGA-elements), which may influence *NRAMP* expression [86]. In rice, the expression of *OsNRAMP5* is regulated by IAA, potentially indirectly affecting metal transport by modulating cell wall Cd-binding capacity and nitric oxide (NO) levels [96]. NO, as a signaling molecule, plays a role in metal detoxification mechanisms, though further research is needed.

ABA is an important stress response hormone [97]. There are multiple ABA-responsive elements (TGA-responsive elements) in the homeopathic elements of the *NRAMP* gene family in tomato and eggplant, indicating that they are directly regulated by ABA [38,85]. ABA may reduce Cd absorption by down-regulating the expression of the *NRAMP* gene in *Arabidopsis* [97]. The same phenomenon has been confirmed in *Arabidopsis* studies, but this regulation is selective. For instance, in *Arabidopsis*, ABA inhibits the expression of *AtNRAMP1*, but has a relatively small impact on other *AtNRAMP* genes [97].

The promoter regions of the *NRAMP* gene family members in tomatoes contain SA response elements (TCA and as-1), indicating that their expression may be regulated by the SA signaling pathway [38]. In addition, the expression of *NRAMP* genes in peanuts and wheat is regulated by the JA signaling pathway, which in turn affects the absorption and distribution of metal ions [93]. The promoters of the wheat *TaNRAMP* gene family contain elements related to hormone responses. Approximately 90% of the *TaNRAMP* genes contain the JA response *cis*-elements TGACG and CGTCA motifs related to defense [68]. These research achievements provide new ideas for regulating the accumulation of metal ions by using endogenous hormones.

In summary, plant hormones influence the function of *NRAMP* genes through transcriptional regulation and signaling pathway interactions, thereby modulating the uptake of essential metals (such as Fe, Mn, Zn, etc.) and plant tolerance to toxic metals (such as Cd, Pb). These findings provide new insights for regulating metal ion accumulation using endogenous hormones. However, the sensitivity of *NRAMP* genes to hormones and stress varies among species. For example, the responsiveness of *BnNRAMP* genes in rapeseed (*B. napus*) to hormones and stress may be relatively low [26], and response patterns differ across various crops.

### 4.2. Regulation via Metal-Binding Sites in Genes

The metal-binding sites of NRAMPs play a critical role in mineral element absorption. Early studies found that in the bacterium *Deinococcus radiodurans*, the methionine residues in NRAMPs make Cd^2+^ a preferred substrate. When M230 is mutated to alanine (Ala), Cd^2+^ absorption decreases significantly, while absorption of Mn^2+^ and Fe^2+^ remains unaffected [44]. Similarly, mutations in these sites in plants can regulate the transport and absorption of metal elements, providing a breeding direction for developing heavy metal-resistant plant varieties. For example, in rice, a mutation at the Q337 site of the eighth transmembrane helix (TM8) of *OsNRAMP5* alters the stability between TM7 and TM8, thereby affecting protein conformation. Under Cd pollution conditions, the Cd and Mn concentrations in the grains of this mutant are reduced by 50% and 30%, respectively, with no impact on yield [54]. Additionally, the M235 site of *OsNRAMP5* may be involved in the selective transport of Mn^2+^ and Cd^2+^. The sulfur-rich properties of methionine play a key role in Cd transport. By mutating these gene sites, the heavy metal transport function of *OsNRAMP5* is abolished, Cd absorption in the roots is inhibited, and Cd concentrations in grains and straw are significantly reduced [55]. In *Arabidopsis*, random mutations in *AtNRAMP4* enable its transport activity for Fe^2+^, Mn^2+^, Zn^2+^, and Cd^2+^, while specific mutations (L67I, L67V, E401K, and F413I) restore Fe^2+^ absorption and reduce Cd sensitivity. Protein phosphorylation also regulates the activity and function of NRAMPs [88]. For example, the N-terminal serine residues S20, S22, and S24 of the *AtNRAMP1* protein in *Arabidopsis* undergo phosphorylation modifications, which alter its subcellular localization, affect Mn absorption, and regulate plant tolerance to Mn toxicity [48]. Clearly, mutations in key gene sites play an important role in metal transport. However, whether these mutations have the same effects in other plants requires further verification.

### 4.3. Transcription-Factor Regulation of NRAMP Genes

The *cis*-acting elements play essential roles in the transcriptional regulation of NRAMP genes. The promoter regions of *NRAMP* genes are enriched in hormone-, stress-, and metal-responsive elements, suggesting that transcription factors may regulate *NRAMP* expression under heavy-metal stress. At present, direct evidence for transcription-factor control of specific *NRAMP* genes is still limited, but several studies indicate that MYB (Myeloblastosis), WRKY, bHLH (basic Helix-Loop-Helix), and ERF (Ethylene Responsive Factor) transcription factors are important components of the broader metal-stress regulatory network. In rice, the MYB transcriptional repressor OsMYBxoc1 reduces Fe and Mn accumulation by directly suppressing the transcription of *OsNRAMP5*, showing that *NRAMP* genes can be transcriptionally controlled by stress-related transcription factors [98].

WRKY transcription factors should be considered in the NRAMP regulatory framework because they regulate plant responses to heavy-metal stress through ROS homeostasis, antioxidant defense, metal chelation, vacuolar compartmentalization, and transporter-related pathways. Recent reviews have emphasized that WRKY transcription factors participate in responses to Cd, As, Cu, and Al stresses and can regulate downstream genes involved in heavy-metal tolerance [99]. Therefore, WRKY factors may indirectly influence NRAMP-mediated metal uptake by coordinating oxidative stress responses and metal detoxification pathways. In tomato, a genome-wide analysis showed that both the WRKY and bHLH transcription factor families are differentially expressed under Cd stress, indicating their potential involvement in Cd-responsive transcriptional networks [100].

bHLH transcription factors are also highly relevant to NRAMP-associated metal homeostasis because many NRAMPs transport Fe and Mn in addition to Cd. In *Arabidopsis*, bHLH121 and clade IVc bHLH transcription factors synergistically regulate Fe homeostasis by activating FIT and clade Ib bHLH transcription factors, thereby stimulating Fe uptake [101]. Earlier work also showed that bHLH121 functions together with bHLH Ivc transcription factors to facilitate FIT activation under Fe deficiency [102]. Since Fe-deficiency responses can activate metal transport pathways that may also transport Cd, bHLH-mediated Fe signaling may indirectly influence NRAMP-associated Cd/Fe/Mn uptake and redistribution. However, direct binding of bHLH transcription factors to *NRAMP* promoters has not been sufficiently demonstrated and should be tested in future studies.

ERF transcription factors connect ET signaling with Cd tolerance. Recent Cd-stress reviews identify ERF, together with WRKY, MYB, bHLH, and bZIP (basic leucine zipper), as one of the major transcription-factor families involved in plant Cd tolerance. ERF proteins regulate Cd tolerance through pathways associated with ROS homeostasis, nitrate metabolism, ET biosynthesis, and ET signaling [98,103]. Although direct binding of ERF proteins to NRAMP promoters has not yet been widely demonstrated, ERF-mediated ET responses may indirectly affect *NRAMP* expression because ET signaling is already linked to NRAMP activity and metal transporter regulation in plants.

In *Arabidopsis*, the transcription factor INO (INNER NO OUTER) directly suppresses the expression of *AtNRAMP1* during early seed germination to prevent Fe overload-induced oxidative damage [104]. The promoter regions of the *SpNRAMP* family in *S. polyrhiza* are rich in MYC motifs and stress-responsive elements, indicating potential involvement in metal-stress responses [92]. Together, these findings suggest that *NRAMP* genes are regulated by a complex transcriptional network involving metal homeostasis, hormone signaling, oxidative stress, and developmental cues.

### 4.4. Post-Translational Regulation of NRAMPs

In addition to transcriptional regulation, post-translational modifications provide a rapid mechanism for controlling NRAMP protein abundance, localization, activity, and degradation under heavy-metal stress. Phosphorylation is currently the best-characterized post-translational modification of plant NRAMPs. In *Arabidopsis*, Mn triggers phosphorylation-mediated endocytosis of AtNRAMP1, thereby reducing AtNRAMP1 abundance at the plasma membrane and helping plants avoid excessive Mn uptake [48]. More recently, a Cd-specific WAKL4–NRAMP1 regulatory module was identified. Under Cd stress, WAKL4 accumulates in roots and phosphorylates NRAMP1 at Tyr488, which promotes NRAMP1 ubiquitination and vacuole-dependent degradation, consequently reducing Cd uptake [87].

Ubiquitination should therefore be considered an important post-translational regulatory mechanism for NRAMP-mediated heavy-metal uptake. By controlling NRAMP stability and vacuolar degradation, ubiquitination can rapidly decrease transporter abundance and restrict toxic metal entry. However, the E3 ubiquitin ligases that recognize phosphorylated NRAMPs remain largely unknown. Identifying NRAMP-specific E3 ligases and determining whether similar phosphorylation–ubiquitination modules exist in crops such as rice, wheat, and tomato will be important for future low-heavy-metal breeding.

SUMOylation may represent another regulatory layer, although direct experimental evidence for the SUMOylation of plant NRAMPs remains lacking. In plants, post-translational modifications such as phosphorylation, ubiquitination, SUMOylation, neddylation, lipidation, and S-nitrosylation regulate protein stability, localization, and function under heavy-metal stress [105]. Therefore, future studies should test whether NRAMPs are SUMOylated under Cd, Mn, Fe, or Zn stress and whether SUMOylation affects their trafficking between the plasma membrane, endosomes, and vacuoles.

### 4.5. miRNA and Post-Transcriptional Regulation of NRAMP Genes

miRNA-mediated regulation provides another important layer of control over NRAMP under heavy-metal stress. miRNAs regulate gene expression mainly by cleaving target mRNAs or inhibiting translation, allowing plants to rapidly adjust metal transporter abundance in response to environmental signals. A clear example has been reported in *B. napus*, in which 22 NRAMP transporter genes were identified, and several were responsive to Cd treatment. Among them, *BnNRAMP1b* was strongly induced by Cd and functionally associated with Cd, Zn, and Mn transport. Degradome analysis showed that BnNRAMP1b can be cleaved by miR167, and the contrasting expression patterns of BnNRAMP1b and miR167 under Cd stress supported post-transcriptional regulation of BnNRAMP1b by miR167 [106].

The miR167–BnNRAMP1 module indicates that NRAMP-mediated heavy-metal uptake can be regulated not only by promoter activity and protein modification, but also by small-RNA-guided transcript cleavage. This mechanism may help plants fine-tune Cd uptake while maintaining essential metal homeostasis. However, only a few NRAMP-targeting miRNA modules have been experimentally validated. Future work should combine degradome sequencing, small-RNA sequencing, 5′ RACE validation, and CRISPR-based editing of miRNA target sites to identify conserved and species-specific miRNA–NRAMP regulatory modules.

### 4.6. Conservation and Divergence of NRAMP Regulatory Mechanisms Between Monocots and Dicots

Comparative studies suggest that several regulatory features of NRAMP transporters are broadly conserved between monocots and dicots, although the strength of experimental evidence differs among species. First, both monocot and dicot NRAMP genes respond to metal availability and heavy-metal stress. In monocots, rice OsNRAMP1, OsNRAMP2, and OsNRAMP5 are regulated by Fe, Mn, Cd, and other metal stresses and participate in Cd/Mn or Fe/Cd transport and redistribution [49,50,52,53]. In dicots, *Arabidopsis* AtNRAMP1, AtNRAMP3, and AtNRAMP4, as well as NRAMP members in tomato, peanut, tobacco, and Brassica species, are also responsive to metal deficiency or heavy-metal exposure [18,48,65,66,78,83]. This indicates that metal-dependent transcriptional regulation of *NRAMP* genes is a conserved regulatory feature across flowering plants.

Second, the nutrient–toxin trade-off appears conserved between monocots and dicots. In rice, OsNRAMP5 functions as a major Cd/Mn transporter, and genetic manipulation of OsNRAMP5 can reduce Cd accumulation but may also affect Mn nutrition [50,52,54]. Similar Cd–Mn or Cd–Fe interactions have been reported in dicot species, where NRAMP transporters participate in the uptake or redistribution of essential metals and toxic analogs [25,78,83]. Thus, the regulatory challenge of reducing Cd uptake while maintaining essential micronutrient homeostasis is not unique to rice but represents a general problem for NRAMP-mediated metal transport in both monocots and dicots.

Third, transcription factor- and hormone-related regulation may be partially conserved, but direct evidence remains limited. In monocots, the MYB transcriptional repressor OsMYBxoc1 directly binds the *OsNRAMP5* promoter and suppresses *OsNRAMP5* transcription in rice [107]. In dicots, the YABBY transcription factor INO suppresses *AtNRAMP1* expression during early seed development, and broader Cd-stress regulatory networks involving MYB, WRKY, bHLH, ERF, and bZIP transcription factors have been described [89,104,107]. These findings suggest that monocots and dicots both use transcriptional networks to regulate NRAMP-mediated metal homeostasis, but the specific transcription factors and target *NRAMP* genes may have diverged.

Fourth, post-translational regulation is well documented in dicots but still poorly characterized in monocots. In *Arabidopsis*, Mn triggers phosphorylation-mediated endocytosis of AtNRAMP1, and Cd induces the WAKL4–NRAMP1 module, in which WAKL4 phosphorylates NRAMP1 and promotes its ubiquitination and vacuole-dependent degradation [48,87]. These mechanisms provide rapid control of transporter abundance at the plasma membrane. Comparable phosphorylation-, ubiquitination-, or trafficking-based regulation has not yet been fully demonstrated for rice OsNRAMPs or other monocot NRAMPs. Therefore, whether dicot-type post-translational regulation is conserved in monocots remains an important open question.

Finally, lineage-specific divergence is also evident. In monocots, especially rice, natural allelic variation, weak alleles, promoter editing, and allele pyramiding of OsNRAMP5 and OsHMA3 have been developed as practical strategies for low-Cd breeding [54,61,108]. In dicots, evidence is stronger for transcriptional responses, miRNA-mediated regulation, and stress-responsive promoter elements, but fewer breeding-oriented NRAMP alleles have been validated [54,106]. Overall, monocots and dicots share conserved NRAMP regulatory principles, including metal-responsive expression, micronutrient–toxin trade-offs, and transcriptional regulation. In contrast, specific upstream regulators, post-translational mechanisms, and breeding applications show lineage-specific differences that require further comparative validation.

## 5. Evolutionary Conservation of NRAMP Transporters Across Green Plants

**Figure 1:**
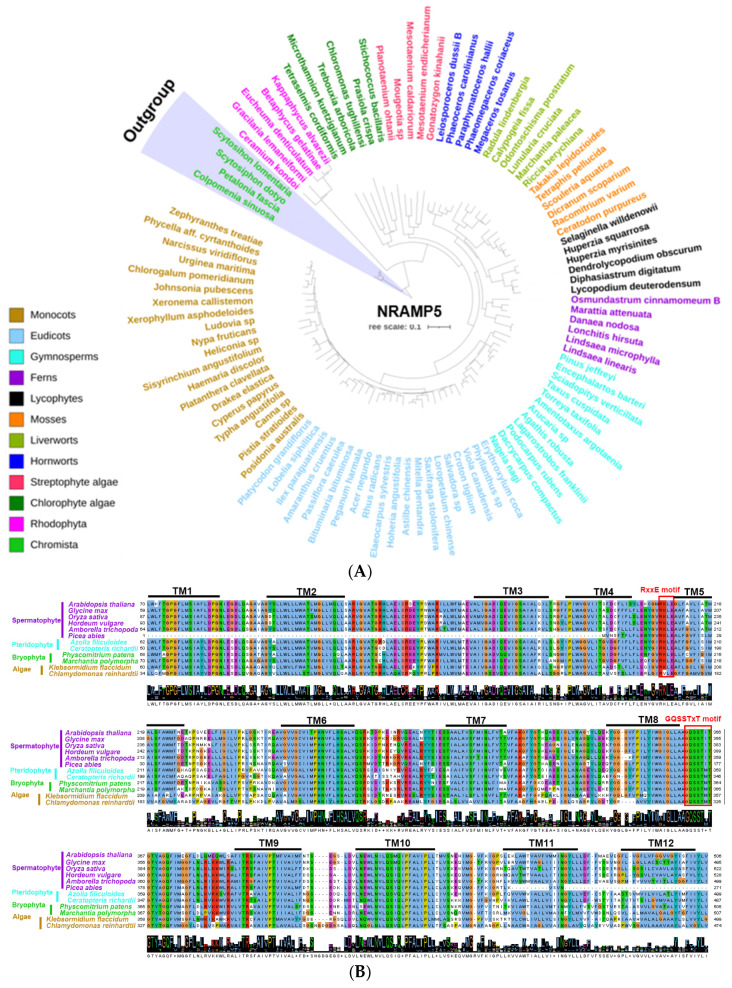
Evolutionary analysis of NRAMPs in land plants and algal species. (**A**) The phylogenetic tree includes species from the major clades of eudicots, monocots, gymnosperms, ferns, lycophytes, mosses, liverworts, hornworts, and algae. Motif alignment (**B**) of NRAMPs in diverse plants.

**Figure 2:**
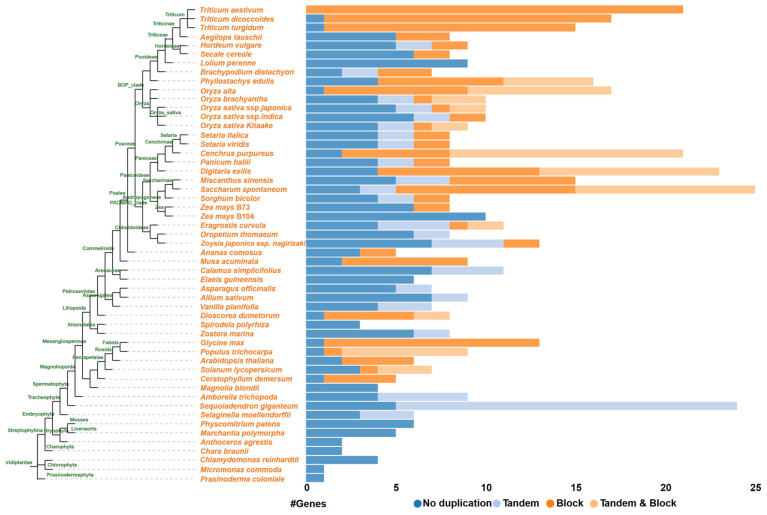
Tandem and block gene duplicate of *NRAMP* gene family in *Chlorophyta* and *Embryophyta*. All gene numbers were downloaded from the PLAZA database (https://bioinformatics.psb.ugent.be/plaza/ [accessed on 29 March 2026)]), which contains >100 plant and algal species. The phylogenetic tree of distinct species was obtained through TimeTree (http://www.timetree.org/ [accessed on 29 March 2026)]).

The NRAMP transporters, which belong to the oligopeptide transporter superfamily, have been extensively studied in plants for their roles in Fe and Mn uptake and transport [33]. According to previous methods [109,110], we performed protein sequence alignment of representative species from algae, mosses, ferns, and seed plants, and found that they all contain 12 highly conserved transmembrane domains (Figure 1B). Moreover, they possess highly conserved RxxE and GQSSTxT motifs that play crucial roles in metal ion transport. Six NRAMP members have been identified in *Arabidopsis*. At the same time, eight have been characterized in rice [24] (Figure 2). More recently, 12 NRAMPs have been identified in *S. italica*, 5 in *K. obovata*, 6 in *Hydrangea macrophylla*, and 11 in *P. trichocarpa* [28,85,111]. The NRAMPs are highly conserved across the entire plant kingdom, typically containing 10 to 12 transmembrane domains [78]. For example, 7–13 putative transmembrane domains were identified in *S. polyrhiza*, 11 in *S. italica*, 12 in *Phaseolus vulgaris*, and 10–12 in *G. max* [23,97,112].

## 6. Expression Analysis of NRAMP Genes in Diverse Plants

**Figure 3:**
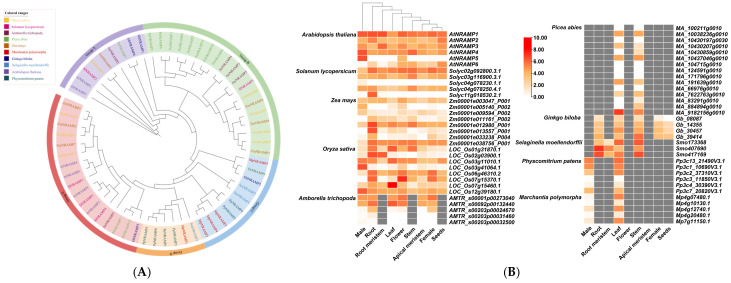
Phylogenetic analysis (**A**) and expression (**B**) of *NRAMP* genes of diverse tissues and organs (male portion, female portion, apical meristem, root meristem, flower, seed, root, leaf, and stem) in monocots (*O. sativa*, *Z. mays*), eudicots (*A. thaliana*, *S. lycopersicum*), basal angiosperms (*A. trichopoda*), gymnosperms (*P. abies*, *G. biloba*), lycophytes (*S. moellendorffii*), moss (*P. patens*), and liverworts (*M. polymorpha*). Expression data was downloaded and from public RNA-seq datasets CoNekT (https://evorepro.sbs.ntu.edu.sg/heatmap/comparative/tree/41304/raw [accessed on 30 March 2026)]).

**Figure 4:**
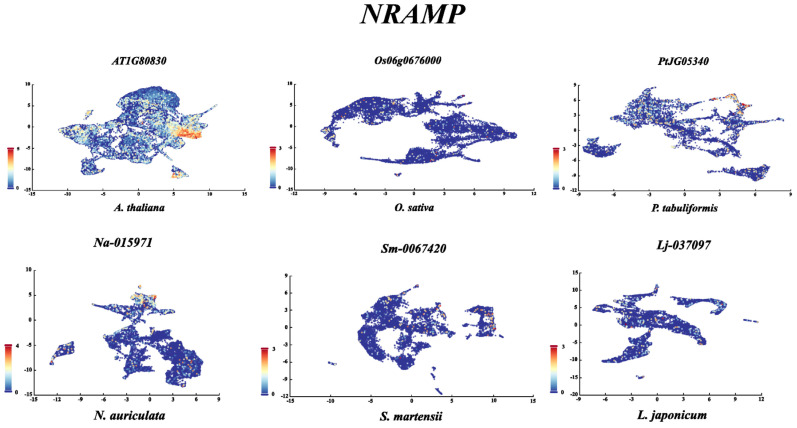
Single-cell expression analysis of *AtNRAMP1* (*A. thaliana*), *OsNRAMP1* (*O. sativa*), *PtJG05340* (*P. tabuliformis*), *Na-015971* (*N. auriculata*), *Sm-0067420* (*S. martensii*), and *Lj-037097* (*L. japonicum*).

We also analyzed the expression profiles for metal transporter homologs across diverse plant species, ranging from ubiquitous to highly tissue-specific (Figure 3). First, we performed a phylogenetic analysis of NRAMP family members from *A. thaliana*, *S. lycopersicum*, *O. sativa*, *Zea mays*, *Amborella trichopoda*, *Picea abies*, *Ginkgo biloba*, *Selaginella moellendorffii*, *Physcomitrium patens*, and *Marchantia polymorpha*, and found that they were relatively evenly distributed among five groups (Figure 3A). Subsequently, we performed tissue-specific expression analysis on these *NRAMP* genes (Figure 3B). In *Arabidopsis*, *AtNRAMP1* stood out for its ubiquitous and high expression across all tissues, peaking in the root meristem and root, suggesting a fundamental role in basal metal homeostasis. In contrast, *AtNRAMP4* showed a strong preference for male tissues, while *AtNRAMP5* is exceptionally male-specific, with virtually no expression elsewhere. *AtNRAMP6* is notable for its peak in seeds. The tomato genes displayed equally distinct patterns. *Solyc11g018530.2.1* was a textbook example of root-specific expression, with high levels in roots and near-absence in all other tissues. Conversely, *Solyc02g092800.3.1* was broadly expressed, with its lowest point in male tissues, suggesting a general metabolic role. In maize, several genes show clear specificity. *Zm00001e013557_P001* was overwhelmingly root-specific, mirroring the tomato root-specific gene. *Zm00001e005140_P002* was primarily expressed in male tissues, while *Zm00001e011161_P002* displayed a reciprocal pattern with highest expression in root and near-silence in reproductive organs. The rice genes provided some of the most dramatic examples of specialization. *LOC_Os07g15460.1* was an extreme case of leaf-specificity, with expression in leaf dwarfing all other tissues. *LOC_Os07g15370.1* peaked massively in flower, the highest single value in the dataset. *LOC_Os03g41064.1* was almost exclusively expressed in male tissues, reinforcing a conserved theme of male-specific *NRAMP-like* genes across species. *LOC_Os02g03900.1* showed a dual root and root meristem specificity, while *LOC_Os01g31870.1* was strongly expressed in vegetative tissues (leaf and stem) but nearly absent in reproduction. Genes from other species enriched these patterns. In *A. trichopoda*, *AMTR_s00092p00132440* peaked in leaf. The spruce gene *MA_9182156g0010* showed very high expression in stem, likely related to vascular development. In the bryophytes *P. patens* and *M. polymorpha*, several genes, such as *Mp7g11150.1*, are highly expressed in stems but nearly absent in male tissues, indicating early evolutionary divergence in tissue-specific regulation. Overall, the dataset captures a fundamental biological principle: while some genes are constitutively expressed to maintain core cellular functions, many others have evolved tight regulatory control for specialized roles in specific organs. The repeated occurrence of root-specific, male-specific, and leaf-specific genes across *Arabidopsis*, tomato, maize, and rice suggests conserved evolutionary pressures and functions for these transporters in mineral nutrition, reproduction, and photosynthesis. Single-cell results showed that the *AtNRAMP1* gene was highly expressed in the examined tissues, while other *NRAMP1* genes displayed low expression (Figure 4).

## 7. Expression Analysis of *NRAMP* Genes in Rice and Arabidopsis in Response to Ion Stress

**Figure 5:**
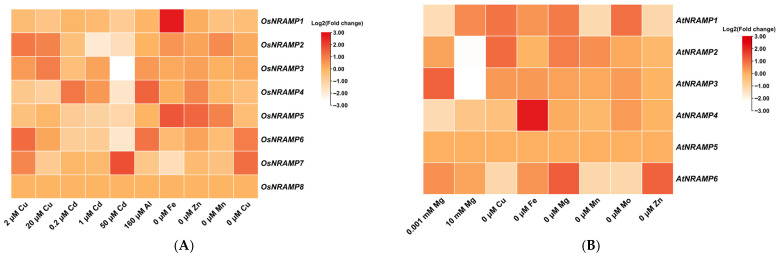
The expression of *NRAMP* genes in rice (**A**) and *Arabidopsis* (**B**) in response to different supply levels of various metal elements (Fe, Mn, Cu, Zn, Cd, Co, Al, Mg). These *NRAMP* gene expression data were obtained from the Plant Public RNA-seq Database (https://plantrnadb.com/ [accessed on 31 March 2026)]).

In addition to the analysis of tissue-specific expression, the responses of *NRAMP* genes to different levels of metal ion stress in rice and Arabidopsis were analyzed (Figure 5). Under excess Cu stress, *OsNRAMP2*, *OsNRAMP3*, *OsNRAMP6*, and *OsNRAMP7* were significantly induced. Notably, *OsNRAMP6* nd *OsNRAMP7* were also upregulated under Cu deficiency. Under Cd stress, the expression of *OsNRAMP5* and *OsNRAMP6* was suppressed across different Cd concentrations (0.2, 1, and 50 μM), whereas *OsNRAMP3* and *OsNRAMP4* were significantly induced at low Cd levels (0.2 and 1 μM) but markedly downregulated at 50 μM Cd. Similarly, *OsNRAMP2* was significantly downregulated at 1 μM and 50 μM Cd. Under excess Al stress, *OsNRAMP4* and *OsNRAMP6* were strongly induced. Under Fe, Mn, and Zn deficiencies, *OsNRAMP5* was consistently upregulated. Interestingly, under Fe deficiency, *OsNRAMP1* and *OsNRAMP7* exhibited opposite expression patterns: *OsNRAMP1* was significantly induced, whereas *OsNRAMP7* was markedly suppressed. Additionally, under Mn deficiency, *OsNRAMP2* was significantly upregulated.

Meanwhile, all six members of the *NRAMP* gene family in Arabidopsis were analyzed [113]. Under low Mg supply, *AtNRAMP2* and *AtNRAMP3* were significantly induced, whereas *AtNRAMP1* and *AtNRAMP4* were markedly suppressed. Under high-Mg supply, *AtNRAMP1* was slightly induced, whereas *AtNRAMP2* and *AtNRAMP3* were significantly downregulated. Under Cu deficiency, *AtNRAMP1* and *AtNRAMP2* were strongly induced, whereas *OsNRAMP6* was slightly repressed. Under Fe deficiency, *OsNRAMP4* was highly upregulated. Under complete Mg deficiency, *AtNRAMP1*, *AtNRAMP2*, and *AtNRAMP6* were significantly induced. Under Mn deficiency, *AtNRAMP1* and *AtNRAMP6* were slightly suppressed. Under Mo deficiency, *AtNRAMP1* was induced, whereas *AtNRAMP6* was repressed. Conversely, under Zn deficiency, the opposite pattern was observed: *AtNRAMP1* was suppressed, while *AtNRAMP6* was induced.

## 8. Conclusions and Future Perspectives

**Figure 6:**
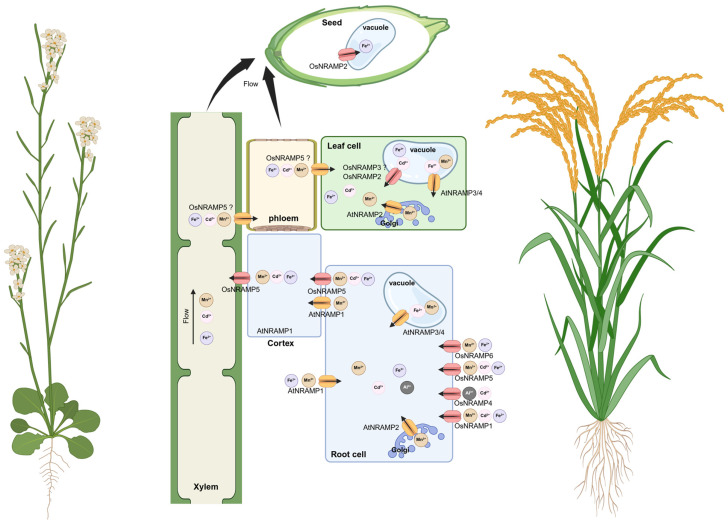
Proposed model of NRAMP-mediated metal uptake, intracellular redistribution, long-distance translocation, and accumulation in rice and Arabidopsis. Plasma membrane-localized NRAMPs mediate root uptake of divalent metals such as Mn^2+^, Fe^2+^, and Cd^2+^, whereas tonoplast-localized NRAMPs participate in vacuolar remobilization and redistribution to developing tissues. Transport activity is further regulated by conserved metal-binding residues, transporter trafficking, phosphorylation, ubiquitination, and stress-responsive transcriptional pathways.

In recent years, research on the functions of plant *NRAMP* family members has advanced significantly, highlighting their critical roles in metal homeostasis and their considerable potential for applications in plant genetic improvement and environmental remediation. This review has systematically summarized the current knowledge regarding the distribution, evolutionary relationships, and functional mechanisms of NRAMP transporters across various plant species. To provide an integrated overview of NRAMP function and regulation, we summarized the major transport routes, representative NRAMP members, tissue-specific expression patterns, and regulatory mechanisms in a schematic model (Figure 6). In this model, plasma membrane-localized NRAMPs such as OsNRAMP1, OsNRAMP5, and AtNRAMP1 mainly contribute to root uptake or cellular influx of divalent metals, including Mn^2+^, Fe^2+^, and Cd^2+^. In contrast, tonoplast-localized NRAMPs such as OsNRAMP2, AtNRAMP3, and AtNRAMP4 participate in vacuolar remobilization and intracellular redistribution of Fe, Mn, and Cd [48,49,53,83]. The model also highlights the nutrient–toxin trade-off because several NRAMP members transport both essential metals and toxic analogs, especially Cd and Mn, making NRAMPs important targets for low-Cd breeding and phytoremediation [25,30,31]. In addition, the model summarizes regulatory inputs from phytohormones, transcription factors, miRNAs, post-translational modifications, and natural or engineered allelic variation, which together determine *NRAMP* expression, localization, protein stability, and metal selectivity [48,87,107,110,114]. With a particular focus on their involvement in the uptake, translocation, and detoxification of both essential (e.g., Fe, Mn) and toxic (e.g., Cd, Pb) divalent metal ions (Figure 6).

Despite this progress, our understanding of the full functional spectrum, precise molecular mechanisms and integrated regulatory networks governing plant NRAMP transporters remains incomplete. In particular, comparative studies between monocots and dicots are needed to determine whether regulatory mechanisms identified in model species, such as OsMYBxoc1-mediated repression of *OsNRAMP5* in rice and phosphorylation–ubiquitination-mediated control of AtNRAMP1 in *Arabidopsis*, represent lineage-specific mechanisms or broadly conserved regulatory modules [48,87,107]. Several key challenges and promising research directions remain. First, future studies should clarify the structural basis of NRAMP substrate selectivity, especially how conserved metal-binding residues, transmembrane-domain flexibility, and proton-coupled conformational changes distinguish essential metals such as Mn and Fe from toxic metals such as Cd and Pb [32,44,47]. Second, the subcellular trafficking of NRAMPs should be investigated in greater detail, because transporter localization at the plasma membrane, tonoplast, endoplasmic reticulum, or endomembrane compartments can determine whether NRAMPs promote root uptake, vacuolar release, long-distance transport, or grain accumulation [49,51,114]. Third, post-translational regulation, including phosphorylation, ubiquitination, endocytosis, and vacuole-dependent degradation, should be integrated into future models of NRAMP function. Recent discoveries such as phosphorylation-mediated AtNRAMP1 endocytosis and the WAKL4–NRAMP1 module indicate that plants can actively limit excessive Mn or Cd uptake by controlling NRAMP protein abundance and stability [48,87]. Fourth, breeding strategies should avoid simple loss-of-function approaches that cause micronutrient deficiency or increase stress sensitivity. Instead, weak alleles, promoter editing, tissue-specific expression modulation, and allele pyramiding with other metal transporters such as *HMA* genes may provide more balanced strategies for reducing toxic metal accumulation while maintaining essential mineral nutrition [49,54,114]. Finally, combining comparative genomics, single-cell transcriptomics, ionomics, structural prediction, genome editing, and field validation will be necessary to translate mechanistic knowledge of NRAMPs into low-heavy-metal crops and efficient phytoremediation systems [41,43]. Finally, although NHX transporters are mainly discussed in the context of salinity and Na^+^/H^+^ homeostasis, recent work on NHX-mediated ion balance provides a useful broader framework for understanding how membrane transporters can be engineered to improve crop resilience under complex soil stresses, including combined salinity and heavy-metal stress [115].

**Table 1:** Strategies for reducing Cd accumulation while avoiding Mn deficiency in NRAMP-related low-Cd breeding.

Strategy	Representative Target/Example	Mechanism	Advantage	Limitation or Caution	References
Complete NRAMP knockout	OsNRAMP5 knockout in rice	Strongly reduces Cd and Mn uptake because OsNRAMP5 is a major root transporter for both metals	Effective reduction in Cd uptake and grain Cd	May reduce Mn accumulation and cause growth, yield, or stress-resistance penalties under low-Mn conditions	[50,52,108]
Weak point mutation	OsNRAMP5-Q337K	Partially reduces OsNRAMP5 transport activity rather than fully abolishing it	Reduces Cd accumulation while avoiding severe Mn deficiency	Cd and Mn transport are both reduced; effect may depend on genotype and soil Mn status	[54,55]
Engineered amino acid substitution	OsNRAMP5-M235A, M235C, A232S + M235A	Alters metal-binding or transport-tunnel properties to reduce Cd transport while retaining Mn transport	Potentially improves Cd/Mn selectivity	Most evidence is from yeast assays; plant and field validation are still required	[116]
Natural allelic variation/tandem duplication	Pokkali OsNRAMP5 duplication	Higher OsNRAMP5 expression increases Cd and Mn uptake into root cells but decreases Cd release to xylem	Natural allele can reduce grain Cd without yield or eating-quality penalty after introgression	Allele may be rare and requires marker-assisted introgression into elite cultivars	[60]
Regulatory-region editing	CRISPR/Cas9 editing of OsNRAMP5 regulatory region	Reduces OsNRAMP5 translation without changing its expression pattern	Lowers grain Cd while maintaining Mn accumulation and agronomic traits	Requires careful validation to avoid excessive reduction in transporter activity	[108]
Tissue- or cell-specific modulation	Tissue/cell-specific editing, knockdown, or re-expression of OsNRAMP5	Attempts to reduce Cd entry or xylem transfer in key root cell layers while preserving Mn uptake in necessary tissues	More precise than whole-gene knockout; may reduce trade-off	Direct validated examples remain limited; should be presented as an emerging strategy	[52,108]
Vacuolar Cd sequestration	OsHMA3 overexpression or functional OsHMA3 alleles	Enhances Cd sequestration into vacuoles, reducing Cd movement to shoots and grains	Can reduce grain Cd without directly impairing Mn uptake	Efficiency depends on OsHMA3 allele, expression level, and genetic background	[89,90]
Allele pyramiding	OsNRAMP5LAA + OsHMA3LAA	Combines reduced Cd uptake/altered OsNRAMP5 trafficking with enhanced Cd sequestration	Reduces grain Cd without Mn-deficiency sensitivity, yield penalty, or heat/low-Mn stress penalty	Needs validation across diverse cultivars and environments	[114]
Agronomic Mn management	Mn fertilization, Mn–lime treatment, water/redox management	Mn competes with Cd uptake and may suppress Cd accumulation through the OsNRAMP5 pathway	Non-transgenic and field-applicable	Excess Mn can cause toxicity; timing, dose, and soil redox status are critical	[80,81,117]

## Data Availability

Our expression data can be found in the public database (https://evorepro.sbs.ntu.edu.sg/heatmap/comparative/family/463/raw, accessed on 6 May 2026). Further inquiries can be directed to the corresponding author.
